# Perturbation of Wound Healing, Cytoskeletal Organization and Cellular Protein Networks during Hazara Virus Infection

**DOI:** 10.3389/fcell.2017.00098

**Published:** 2017-11-21

**Authors:** Andrea Molinas, Maria V. Turkina, Karl-Eric Magnusson, Ali Mirazimi, Elena Vikström

**Affiliations:** ^1^Department of Clinical and Experimental Medicine, Faculty of Medicine and Health Sciences, Linköping University, Linköping, Sweden; ^2^Department of Laboratory Medicine, Karolinska Institute, Stockholm, Sweden

**Keywords:** epithelial barrier, homeostasis, epithelial migration, aquaporin 6, IQGAP1, cellular proteome, virus infection

## Abstract

Normal epithelial and endothelial renewal and healing after bacterial and viral challenges are essential for homeostasis along the intestine and the blood and lymphatic vessels. We thus investigated whether and how virus affects migration of human epithelial cells and specifically how the nucleocapsid protein (N) modulates the cellular proteome and interactome using human Caco-2 cells in a wound-healing assay with Hazara virus as a model. Here, Hazara virus blocked cell migration in a dose- and time-dependent manner, disrupted the actin cytoskeleton and specifically reduced the expression of the IQ-motif-containing GTPase-activating protein 1 (IQGAP1) and water channel aquaporin 6 (AQP6) that regulate cytoskeletal organization, water homeostasis and vesicle communication. Moreover, in the Caco-2 cell proteome, we identified several distinct groups of molecules associating with N upon Hazara virus infection, being involved in the ensemble of important cellular processes, e.g., chaperone activity, metabolism, cellular defense against infections, cell morphology, and migration. These events do not only facilitate the virus life cycle, but they are also crucial for membrane and cytoskeleton dynamics, cellular self-renewal and wound healing, being so essential for body integrity and homeostasis.

## Introduction

**Figure 1:**
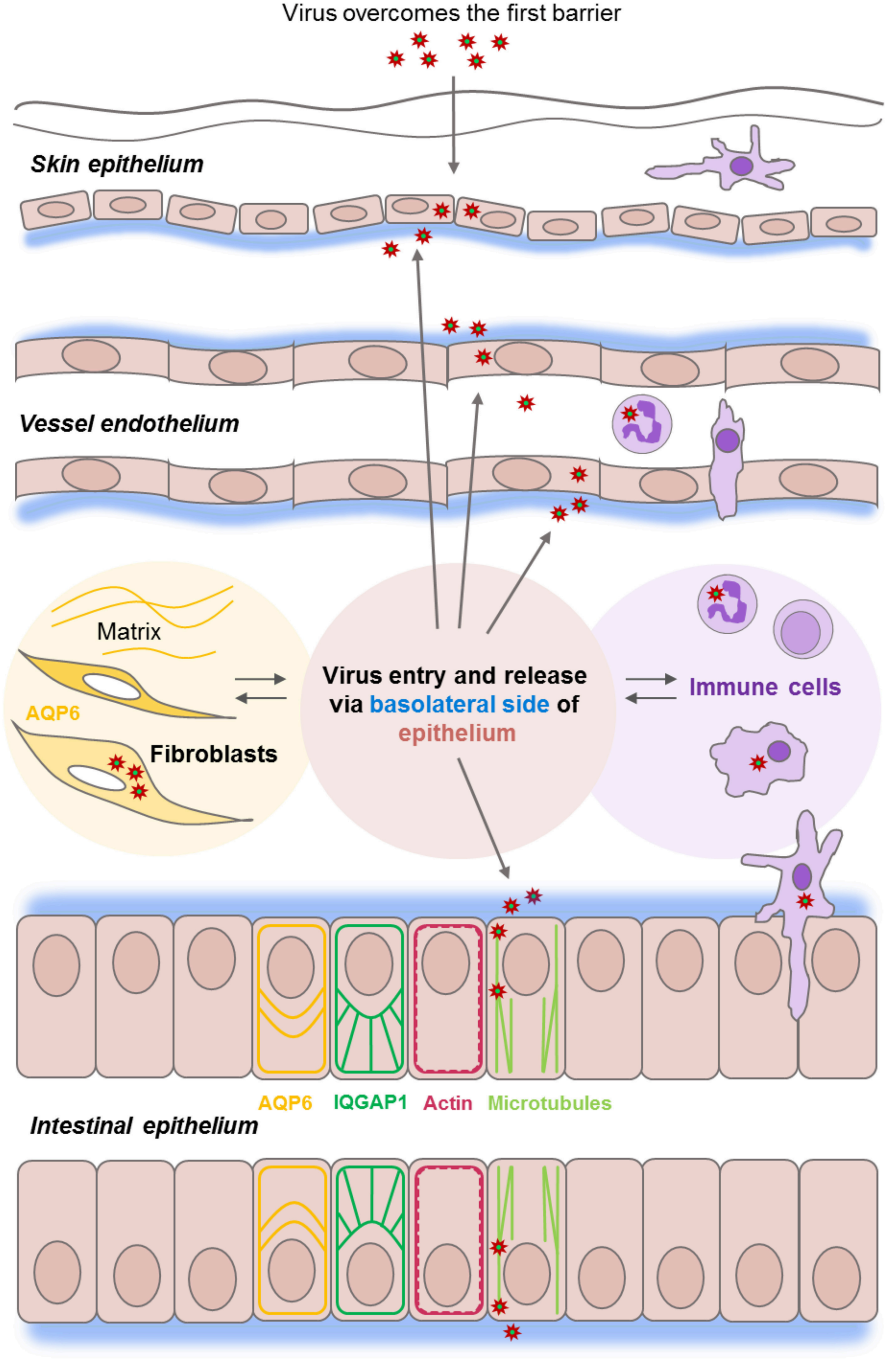
Epithelial barrier functions at the front line of viral infections. Epithelial and endothelial cell monolayers (in pink) provide a series of host barriers to pathogens and other environmental agents. These possess both physical and immune (in lilac) properties. Epithelium constantly moving, renewing, and normally undergoes a wound-healing process which is controlled by an interplay between matrix, water homeostasis (in yellow), and cytoskeleton and junctions-associated proteins (ex. IQGAP1 in green). Viruses of the *Nairovirus* genus (red asterisk) can overcome the first host barrier to enter the body and access underlying cells. Viruses enter host cells by endocytosis, replicates, and assembles in the perinuclear regions of the cytoplasm and leaves the epithelial cell monolayers from the basolateral side (in blue), and further utilize microtubule (light green) and actin (red), the components of host cytoskeleton during the whole life cycle.

Epithelial cells are positioned strategically to provide barriers to pathogens and other environmental agents. They are located both on the outside, e.g., in the skin surface, and on the inside, e.g., along the gastrointestinal tract (Ivanov et al., 2010). The linings of blood and lymphatic vessels of circulatory system are accordingly covered by a specialized form of epithelium, the endothelium (Rodrigues and Granger, 2015). These barriers display both physical and immune characteristics, where the former are potentiated by epithelial cell-to-cell junctions that prevent passage of pathogens and large molecules, and the latter are maintained via detection of antigens and recruitment for instance of phagocytes to the site of infection, potentially resulting in inflammation and tissue damage (Figure 1; Condeelis and Pollard, 2006; Ivanov et al., 2010; Rodrigues and Granger, 2015). The epithelial cell barrier consists of a monolayer of cells that are constantly moving and renewed normally every 72 h in the gut. This is controlled by a highly sophisticated interplay between the cytoskeleton, intercellular junctions, extracellular matrix, surface receptors, signal mediators and fluxes of solutes and water (Ivanov et al., 2010; Rodrigues and Granger, 2015; Friedl and Mayor, 2017). Extensive remodeling of the cytoskeleton is regulated by the Rho family of small GTPases (Zegers and Friedl, 2014), where the IQ-motif-containing GTPase-activating proteins IQGAP scaffolds a plethora molecules to control diverse cellular processes, including cytoskeletal dynamics, cell migration, cell proliferation and vesicle trafficking (Karlsson et al., 2012; Hedman et al., 2015). We have thus provided an evidence that water fluxes through aquaporins (AQP) play a pivotal role in cell migration (Loitto et al., 2009; Karlsson et al., 2013), besides selectively facilitating the transport of water and small uncharged solutes like glycerol both over the cell membrane (Verkman, 2005; Benga, 2012) and intracellular membranes (Molinas et al., 2016). By such interplay with the cytoskeleton and signaling cascades, the AQP do assist directly and indirectly distinct processes, such as cell volume, signal transduction, metabolism, cell migration, and organelle physiology (Saadoun et al., 2005; Verkman, 2005; Loitto et al., 2009; Karlsson et al., 2013; Holm et al., 2016; Molinas et al., 2016).

Viruses can be inhaled or ingested as free viruses, be contained in droplets shed from an infected host, or be injected via arthropods. When they have overcome the first host barrier and enter the body, they get access to underlying permissive cells and can establish an infection (Figure 1).

Some of these arthropod-borne viruses belong to the *Nairovirus* genus of the *Bunyaviridae* family and include pathogens that cause infectious diseases in both animals and humans. The most well-known among them, Crimean-Congo hemorrhagic fever virus (CCHFV) is a human high-fatality rate pathogen causing fever, hemorrhagic symptoms and gastro-intestinal disorders, such as nausea, vomiting, and diarrhea (Ergonul, 2012; Bente et al., 2013). It is an enveloped virus, with three negative-stranded RNA, designated as small, medium and large, which encode the nucleocapsid protein (N), surface glycoproteins, and a RNA-dependent polymerase, respectively (Morikawa et al., 2007). The N is a multifunctional structural protein that plays a key role in the biology of RNA viruses, as it binds to the viral RNA genome and forms a ribonucleoprotein in mature virions. It also interacts with host cell proteins to facilitate the virus life cycle (Wulan et al., 2015), and localizes in the perinuclear region (Andersson et al., 2004) and in the nuclei of infected cells (Cohen et al., 2011). Since N is present in high amounts early during infection, it is useful for clinical diagnosis (Dowall et al., 2012b).

Working with the extremely pathogenic CCHFV requires a high biosafety level 4 laboratory, which has limited the research on specific virus-host interactions and development of antiviral therapies (Whitehouse, 2004; Dowall et al., 2015). The related Hazara virus is classified as a hazard group 2 pathogen and it is not associated with serious diseases in humans, although infections of interferon-knockout mice with either Hazara virus or CCHFV result in similar disease progression (Dowall et al., 2012a). Hazara virus and CCHFV exhibit about 70 and 86% nucleotide and amino acid sequence similarity, respectively (Honig et al., 2004), correlating well with virus serology and pathogenicity. Hazara virus has thus been used as a valid alternative experimental model for a CCHFV infection (Ergonul, 2006; Dowall et al., 2012a), enabling the investigation of *Nairovirus* and the development of antivirals without having access to a biosafety level 4 laboratory.

Our previous investigations have shown that Hazara virus and CCHFV enter host cells by endocytosis, replicate and assemble in the perinuclear regions of the cytoplasm and leave the epithelial cell from the basolateral side (Figure 1; Connolly-Andersen et al., 2007), and further utilize microtubule and actin, the key components of host cytoskeleton during the whole life cycle (Simon et al., 2009). Here, the cytoskeleton regulator and scaffold protein IQ-motif-containing GTPase-activating protein 1 (IQGAP1) seems essential for virus virulence and completion of invasion, replication and egress in some viruses including Ebola, Moloney murine leukemia and swine fever (Hedman et al., 2015). We also showed recently that aquaporin 6 (AQP6), a water transporter and cytoskeleton interactor linked to an intracellular anion channel and involved in vesicle trafficking and sorting (Beitz et al., 2006; Nozaki et al., 2008), seems to have a protective role against Hazara virus infections (Molinas et al., 2016) (Figure 1, shown in yellow).

The aim of this study was to assess in greater detail consequences of virus-host cell interactions, focusing on whether and how the virus infection impacts wound-healing, cytoskeleton organization, IQGAP1 and AQP6 characteristics, and the cellular protein interactome associated specifically with the viral N. To achieve this, we used a Caco-2 epithelial cell migration assay, immunofluorescence imaging, immunoprecipitation, proteomics and bioinformatics. We found here that the virus load and infection duration strongly impacted on epithelial cell structure, signaling and the repair potential.

## Materials and methods

### Epithelial cell culture

Human epithelial colorectal adenocarcinoma Caco-2 cells (86010202 obtained directly from Sigma Aldrich, St. Louis, MO) were grown in Dulbecco's modified Eagle's medium (DMEM) supplemented with 10% heat-inactivated fetal calf serum, 100 U/ml penicillin, 100 μg/ml streptomycin, 1% non-essential amino acids and 2 mM L-glutamine (Life Technologies, Grand Island, NY) at 37°C in 5% CO_2_. This was done for 7–10 days to allow the cells to become mature, differentiated and establish polarized epithelial monolayers.

### Virus stock

The stock for Hazara virus strain JC280 (GenBank accession number M86624.1) was produced in human adrenal cortex adeno carcinoma SW-13 cells (CCL-105 obtained directly from American Type Culture Collection, Manassas, VA) maintained in Leibovitz's L15 medium (L15) supplemented with 10% heat-inactivated fetal bovine serum, 25 mM HEPES, 100 U/ml penicillin, 100 μg/ml streptomycin (Life Technologies) at 37°C in 5% CO_2_.

### Virus infection

Caco-2 cell monolayers were starved in serum-free DMEM overnight and then infected with Hazara virus at three multiplicities of infection (MOI) 0.02, 1 and 2 for 1 h at 37°C in 5% CO_2_ in DMEM supplemented with 2% heat-inactivated fetal bovine serum, 100 U/ml penicillin, 100 μg/ml streptomycin. After 1-h infection, cells were rinsed and maintained for 24 or 48 h post infection (hpi) at 37°C in 5% CO_2_ in serum free DMEM supplemented with 100 U/ml penicillin, 100 μg/ml streptomycin. Then, the cells were processed for migration assay, imaging, and proteomics.

### Migration assay

**Figure 2:**
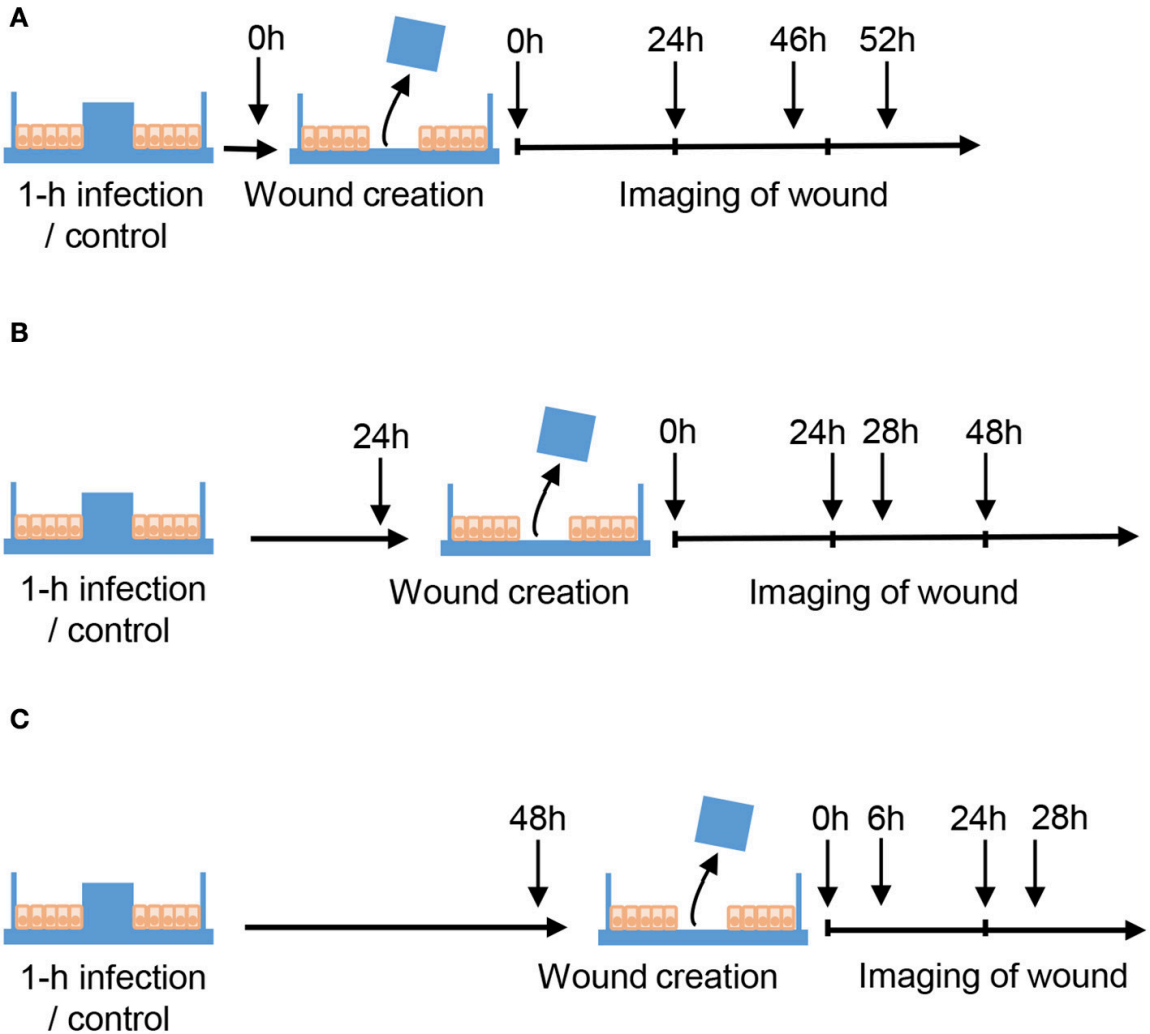
Schematic layout of wound healing assays. Caco-2 cells were cultured in μ-dishes with Ibidi inserts until monolayers were confluent. Cells were then either untreated (Control) or infected with Hazara virus at different MOI for 1 h. After 1-h infection, the cells were rinsed and maintained for 0, 24, or 48 hpi as shown in **(A–C)** respectively. To create a wound, the inserts were removed at 0, 24, or 48 hpi, and the cells were thereafter allowed to migrate. To monitor the wound healing, the images of cells migrating into the gap area of the wound were taken at indicated time using the benchtop microscope.

Caco-2 cells were seeded in μ-dishes with inserts (Ibidi GmbM, Martinsried, Germany) and cultured for 10–12 days to allow the cells to become 100% confluent, mature, differentiated and polarized epithelial monolayers. Then, cells were serum-starved overnight and either non-infected or infected with Hazara virus at MOI 0.02 and 2.0 for 1 h at 37°C in 5% CO_2_ in serum free DMEM supplemented with 100 U/ml penicillin, 100 μg/ml streptomycin. After 1-h infection, the cells were rinsed and maintained for 0, 24, or 48 hpi (Figure 2). At 0, 24 and 48 hpi, the insert was removed to get two cell patches with a 500 ± 50 μm cell-free gap in between allowing the cells to migrate and heal the wound. For each dish, the images of cells migrating into the gap area were taken at indicated time points (Figure 2) using the benchtop microscope JuLI (NanoEnTek Inc., Seoul, South Korea); between imaging, cells were returned to the incubator. Migration activity was calculated by measuring the relative area of the image occupied by cells on each dish at each time point with the Image J software (NIH, Bethesda, MD https://imagej.nih.gov/ij/). At least 3 independent experiments were performed on separate days on different cell passages.

### Determination of virus with immunofluorescence microscopy

Determination of the concentration of Hazara virus in the virus stock and in the monolayers after migration experiments was performed as described previously (Andersson et al., 2004). Briefly, SW-13, or Caco-2 cells in 96-well plates were infected with 10-fold serial dilutions of the virus stock for 1 h at 37°C in 5% CO_2_. Alternatively, Caco-2 monolayers in μ-dishes after closure of the wound were used. Cells were fixed, permeabilized, labeled with polyclonal rabbit anti-CCHFV/Hazara N antibodies (Andersson et al., 2004) and Alexa Fluor 488-conjugated polyclonal goat anti-rabbit antibodies (A11008, Life Technologies). Fluorescent foci were counted in an Axiovert 35 fluorescence microscope (Carl Zeiss, Jena, Germany) equipped with a ProgResC camera (Jenoptik, Jena, Germany). This allowed the determination of the infectious virus titer in focus forming units per ml (FFU/ml).

### Laser scanning confocal imaging

Caco-2 monolayers grown on glass coverslips (thickness 0.17 ± 0.01, 13 mm-diameter; Karl Hecht Assistent, Sondheim, Germany) were washed with PBS pH 7.3 and pretreated with 0.2% Triton X-100 (Sigma Aldrich) in PBS for 2 min on ice. The samples were then fixed in 2.5% paraformaldehyde (Sigma Aldrich) in PBS pH 7.3 for 20 min at room temperature. The pre-treatment with 0.2% Triton X-100 was important for a clear labeling of proteins. After washing with PBS cells were further permeabilized in 0.05% Triton X-100 in PBS for 5 min and washed again. Non-specific background staining was blocked for 10 min in PBS containing 1% BSA and 10 mM glycine. The washing was repeated, and polyclonal rabbit or monoclonal mouse antibodies against CCHFV/Hazara N (Andersson et al., 2004), monoclonal mouse antibodies against IQGAP1 or polyclonal rabbit antibodies against AQP6 (05-504, AB3073, Millipore, Temecula, CA), diluted 1:200 in blocking buffer were then applied for 1 h at 37°C in a moist chamber. After washing, Alexa 568-conjugated goat anti-rabbit and Alexa 488-conjugated goat anti-mouse antibodies (A11036, A11029, Life Technologies) diluted 1:400 were added and incubated for 1 h at 37°C in a moist dark chamber. To detect F-actin, cells were stained with Alexa 488-conjugated phalloidin (A12379, Life Technologies). Nuclei were labeled with DAPI (Life Technologies), according to the manufacturer's instructions. Finally, coverslips were mounted in ProLong Gold (Life Technologies). The specimens were examined through 63x oil immersion objectives with NA 1.40 in a fluorescence microscope Zeiss Axio Observer Z1 with the confocal system Zeiss LSM700 and Zeiss ZEN software (Carl Zeiss, Jena, Germany). Fluorescence intensity in juxta-membrane regions in the cell monolayers and intensity profile plots across the cell monolayers were measures and quantified using the ImageJ software (NIH). At least 3 independent experiments were done on separate days on different cell passages.

### Total cell lysates, SDS-PAGE and immunoblotting

Caco-2 monolayers grown on 6-well plates or flasks were washed with PBS, pH 7.6 and lysed with ice-cold RIPA buffer (150 mM NaCl, 1% deoxycholic acid sodium salt, 1% N-40, 0.1% SDS, 10 mM EDTA pH 8.0, 10 mM Tris pH 7.4 dissolved in PBS) supplemented with 25U nuclease (Thermo Scientific, Rockford, IL), 1 mM phenyl-methyl-sulfonyl-fluoride, 1 mM Na_3_VaO_4_, 25 mM NaF (Sigma Aldrich), protein inhibitors Complete (Roche Diagnostics, Mannheim, Germany). Cell suspensions were homogenized through a 21-gauge needle and centrifuged at 18,000 g for 30 min at 4°C, and the supernatants were collected. The protein concentration in cell lysates was measured with the Bio-Rad D_C_ protein assay (Bio-Rad Laboratories, Hercules, CA). The samples were further diluted in Laemmli sample buffer at equal protein concentrations, heated for 5 min at 95°C and then subjected to electrophoresis. They were loaded on 8–16% SDS-polyacrylamide gels (Lonza, Rockland, ME), and after separation, proteins were electrophoretically transferred to a PVDF Immobilon-FL membrane (Millipore); the quality of the transfer was controlled by Ponceau S staining (Sigma Aldrich). Non-specific binding was blocked by 1-h incubation in 5% non-fat milk in PBS, pH 7.6 containing 0.18% Tween 20 at room temperature. The membranes were then incubated with antibodies against CCHFV/Hazara N (Andersson et al., 2004), IQGAP1, AQP6, and GAPDH (05-504, AB3073, MAB374 Millipore) diluted 1:1000 in blocking buffer overnight at 4°C. After washing, they were treated for 1 h at room temperature with IRDye 800CW goat anti-rabbit or IRDye 680CW goat anti-mouse antibodies (926–32,211, 926–68,070, LI-COR Biosciences, Cambridge, UK), diluted 1:10,000 and washed extensively. The signals were detected and the density ratio was quantified using the Odyssey CLx and the Image Studio software (LI-COR). At least 4 independent experiments were performed on separate days on different cell passages.

### Immunoprecipitation

Total-cell lysates were pre-cleared for 30 min at 4°C with of protein G-Sepharose “4 fast flow” (GE Healthcare, Uppsala, Sweden) and centrifuged before the protein concentration in the supernatants was determined with the Bio-Rad D_C_ protein assay. Samples with equal protein concentrations were then precipitated overnight at 4°C with 1 μg antibodies against CCHFV/Hazara N (Andersson et al., 2004). Immune complexes were captured at 4°C overnight using protein G-Sepharose “4 fast flow.” The beads were collected by pulse centrifugation and washed three times with cold PBS, pH 7.6. Sepharose beads were re-suspended in Laemmli sample buffer, boiled for 5 min at 95°C, collected by centrifugation, and the supernatant was subjected to 8–16% SDS-PAGE (Lonza). The gels were stained with Coomassie Blue (Thermo Scientific). At least 4 independent experiments were done on separate days on different cell passages.

### Protein identification by in-gel digestion and LC-MS/MS

Coomassie Blue-stained protein bands were excised, reduced, alkylated and digested as described previously (Shevchenko et al., 2006); generated peptides were dried, dissolved in 0.1% (v/v) formic acid in water and analyzed by LC-MS/MS. Peptides were separated by reverse phase chromatography on a 20 mm × 100 μm C18 pre column followed by a 100 mm × 75 μm C18 column with particle size 5 μm (NanoSeparatoons, Nieuwkoop, Netherlands) at a flow rate 300 nL/min. EASY-nLC II (Thermo Scientific) by gradient of 0.1% formic acid in water (A) and 0.1% formic acid in acetonitrile (B) as follows: 0–30% B in 50 min, 30–100% B in 40 min. Automated online analyses were performed with a LTQ Orbitrap Velos Pro hybrid mass spectrometer (Thermo Scientific) with a nano-electrospray source.

### Database searching

Raw files were analyzed using Sequest HT in Proteome Discoverer (Thermo Fisher Scientific, San Jose, CS version 1.4.0.288) against a Uniprot Human database available at UniProtKB website (http://www.uniprot.org/taxonomy/9606) with the following parameters: trypsin as a digestion enzyme; maximum number of missed cleavages 2; fragment ion mass tolerance 0.60 Da; parent ion mass tolerance 10.0 ppm; fixed modification, carbamidomethylation of cysteine; variable modifications, methionine oxidation.

### Data evaluation and label-free quantification

Identified proteins were validated using SCAFFOLD (Version 4.4.8; Proteome Software Inc., Portland, OR). Identifications were based on a minimum of 2 unique peptides, minimum 80% peptide identification probability (using the Scaffold Local FDR algorithm), and minimum 95% protein identification probability (using the Protein Prophet algorithm (Nesvizhskii et al., 2003), resulting in a 0.0% decoy FDR). Proteins that contained similar peptides and which could not be differentiated based on MS/MS analysis alone were grouped to satisfy the principles of parsimony. The label-free quantitative analysis of peptides was performed by spectral counting analysis using normalized spectral abundance factor (NSAF) calculated for each protein to normalize run-to-run variations (Zybailov et al., 2006), and quantitative differences were statistically analyzed by two-tailed Student's *t*-test. Differences with *P* < 0.1 were considered statistically significant. Identified proteins were categorized according to gene ontology terms.

### Bioinformatics analysis

The interactions between the identified cellular proteins were analyzed by the Search Tool for the Retrieval of Interacting Genes and Proteins, STRING 10.0 (http://www.ncbi.nlm.nih.gov/pubmed/18940858) using medium confidence score 0.4 and all active interaction sources (Franceschini et al., 2013). STRING and NCBI GO annotations by SCAFFOLD analyses were used to group proteins into functional classes.

### Statistical analysis

Data in the graphs are presented as mean ± SE. Statistical analyses are based on two-tailed Student's *t*-test. The numbers (*n*) are specified in the figure legends. *P* < 0.05 (^*^), < 0.01 (^**^), and < 0.001 (^***^) were considered significant. The experiments on migration and imaging were done at least 3 times on separate days on different cell passages. The experiments for immunoblotting, immunoprecipitation, proteome and interactome were repeated 4 times on separate days. Data evaluation and quantification for proteome and interactome experiments are described above.

## Results

### Hazara virus modulates migration and wound-healing capacity of epithelial cell monolayers

**Figure 3:**
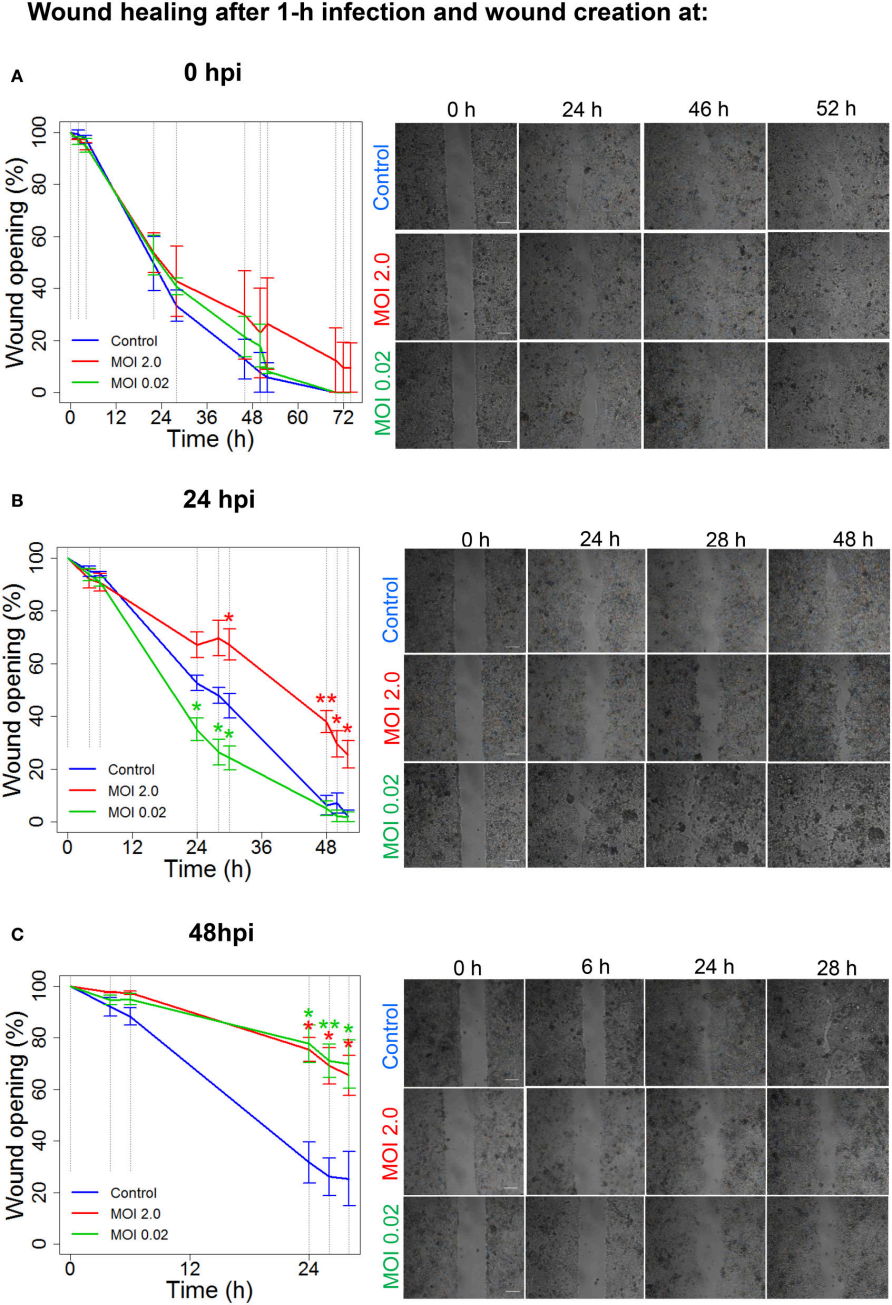
Hazara virus modulates wound healing. Wound healing assays were performed as shown in Figure 2. Cell monolayers were either untreated (Control) or infected with Hazara virus at MOI 2.0 and 0.02 for 1 h. After 1-h infection, the cells were rinsed and maintained for 0, 24, or 48 hpi, as in **(A–C)** respectively. To create a wound, the inserts were removed at 0, 24, or 48 hpi, and the cells were thereafter allowed to migrate as shown in images in in the right panels in **(A–C)**, respectively. The graphs in the left panels represent quantification of wound opening, shown as percent (%) of the original gap. Values are the mean ± SE based on three independent experiments performed on separate days from different cell passages (*n* = 3). Significant differences were analyzed by Student's two-tailed *t*-test and are indicated with ^*^ or ^**^ when *P* < 0.05 or *P* < 0.01 compared to control. Representative images of one of three independent experiments are shown in panels on the right. Bar 200 μm.

When viruses target and enter epithelial cells (Connolly-Andersen et al., 2007), a further perturbation of cell properties and wound-healing process may occur. Therefore, we investigated whether an infection with Hazara virus affected epithelial cell migration, using Ibidi wound-healing chambers (Figure 2). The mature differentiated and polarized epithelial monolayers were infected with Hazara virus at two multiplicities of infection (MOI), 0.02 and 2.0 for 1 h, and wounds were then created at 0, i.e., immediately, or at 24 and 48 h post infection (hpi), allowing the cells to migrate and heal the wound for up to 52 h (Figures 2, 3). For the 0-hpi wound, we observed that the migration rates of virus-infected cells were similar to the control, i.e., untreated cells (Figure 3A). For the wounds created at 24 hpi, the rates of healing for cells infected with Hazara virus at MOI 2.0 were significantly suppressed at 30–52 h. By contrast, the MOI 0.02 resulted in significantly promoted migration between 24 and 30 h after creation of wounds, as compared to the untreated control (Figure 3B). When the wounds were created at 48 hpi, the migration rates of cells infected with Hazara virus at both MOI 0.02 and 2.0 and 0.02 were significantly inhibited around 24–28 h (Figure 3C); the control non-infected wounds were closed at that time and for this reason the study was not continued longer. In this set of experiments, no signs of Caco-2 cell death or apoptosis were seen.

To conclude, Hazara virus can perturb the normal physiology of epithelial cell monolayers and modulate their migration and wound-healing capacity in a dose- and time-dependent manner.

### Hazara virus infection reduces the expression of cytoskeleton-associated proteins

**Figure 4:**
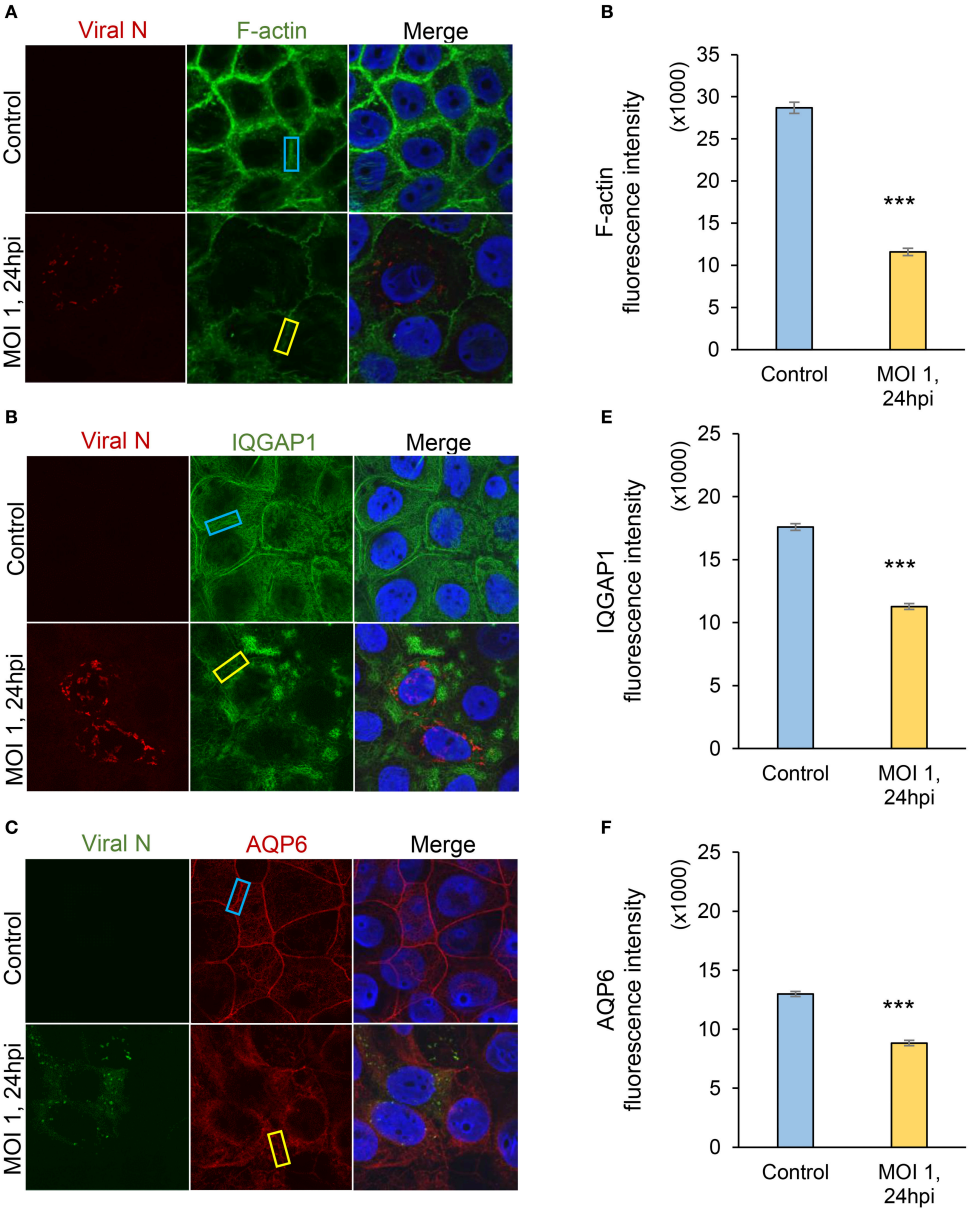
Visualization of Hazara virus, F-actin, IQGAP1 and AQP6 in epithelial cells. Caco-2 cell monolayers were infected with Hazara virus at MOI 1 for 1 h and maintained for 24 hpi. Untreated non-infected Caco-2 cell monolayers were used as a control. Samples were then fixed and stained for: **(A)** viral N (red) and F-actin (green); **(B)** viral N (red) and IQGAP1 (green); **(C)** viral N (green) and AQP6 (red); nuclei were labeled with DAPI (blue). Samples were analyzed by confocal microscopy. The data is from one representative of three independent experiments. Image size is 67.6 × 67.6 μm and pixel size is 0.13 μm. Quantification of fluorescence intensities for F-actin **(D)**, IQGAP1 **(E)** and AQP6 **(F)** measured in juxta-membrane regions in cell monolayers as indicated by blue (untreated control) and yellow rectangles (virus-infected cells, MOI 1, 24 hpi) in **(A**–**C)**. Values in graphs represent the mean ± SE based on 3 independent experiments and 60–63 cells for each condition (same color code as in images). Significant differences were analyzed by Student's *t*-test and are indicated with ^***^ when *P* < 0.001 compared to control.

**Figure 5:**
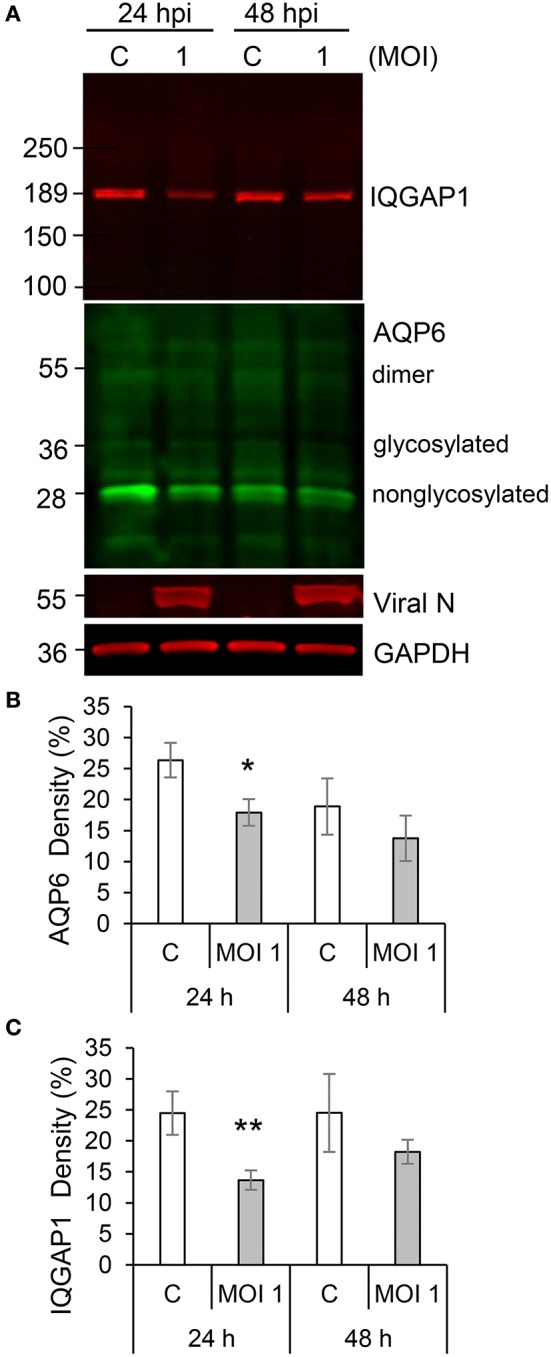
Hazara virus affects the level of IQGAP1 and AQP6 in epithelial cells. **(A)** Cells were untreated controls (c) or infected with Hazara virus at MOI 1 for 1 h and maintained for 24 and 48 hpi. Immunoblots for IQGAP1, AQP6, viral N, and GAPDH. The blots are from one representative out of four independent experiments: **(B,C)** Quantification of blots. AQP6 and IQGAP1 levels normalized to the GAPDH control are indicated as percent (%). Values are the mean ± SE based on four independent experiments performed on separate days from different cell passages (*n* = 4). Significant differences were analyzed by Student's *t*-test and are indicated with ^*^ or ^**^ when *P* < 0.05 or *P* < 0.01 compared to control.

Individual and collective cell migration and restitution of epithelium are driven by actin cytoskeleton reorganization, primarily by dynamic polymerization of monomeric G-actin to F-actin (Ivanov et al., 2010; Friedl and Mayor, 2017) and are regulated by an ensemble of interacting proteins, including IQGAP1 (Karlsson et al., 2012; Hedman et al., 2015) and water fluxes via AQP (Karlsson et al., 2013), which facilitate F-actin formation. Using confocal imaging, we disclosed dramatic decreases in F-actin as well as redistribution of IQGAP1 and AQP6 in Caco-2 monolayers infected with Hazara virus at MOI 1 for 24 h, in clear contrast to control cells (Figures 4A–C). The distribution of these proteins in juxta-membrane regions went from a distinct uniform to a more disorganized and diffuse pattern; AQP6 re-localized to the cytoplasm; IQGAP1 formed cytoplasmic aggregates; and this was observed in more or less all cells in different fields of views. The quantification of fluorescence intensities of F-actin, IQGAP1 and AQP6 in juxta-membrane regions revealed a pronounced and significant decrease of intensity in virus-infected cells (Figures 4D–F), suggesting that cell-to-cell contacts and junctional associations are indeed affected. To further quantify, intensity profile plots across the cell monolayers were measured (Figures S1–S4). Here, non-infected controls displayed pronounced distinct assemblies of F-actin, IQGAP1 and AQP6 at cell-to-cell contacts, whereas the virus-infected cells had clearly more smooth profiles. Viral N was observed in the perinuclear region of infected cells (Figures 4A–C), which is in line with an earlier report (Andersson et al., 2004). Next, we investigated the effect of Hazara virus on the expression levels of IQGAP1 and AQP6 in epithelial cells with immunoblotting (Figure 5A) and further quantification of the density of the bands (Figures 5B,C). Here, the infection with Hazara virus at MOI 1 for 24 h caused a significant decrease in the expression level of either protein.

Thus, the Hazara virus infection resulted in less cytoskeletal F-actin, altered distribution and reduced expression of IQGAP1 and AQP6 appealing for perturbed organization of the cytoskeleton.

### Alterations in the human cellular interactome of the Viral N

**Figure 6:**
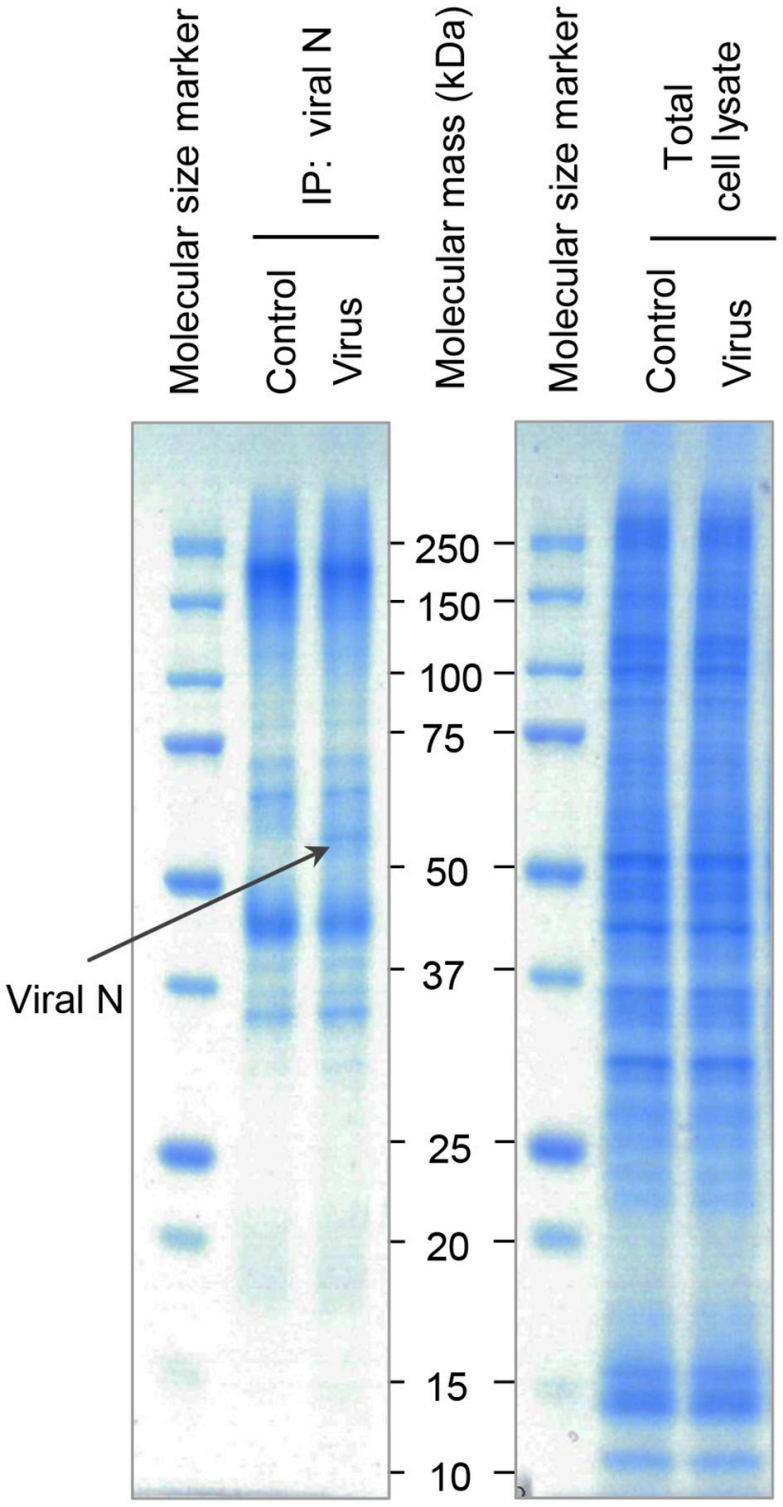
SDS-PAGE analysis of N-associated immune complexes of epithelial cells. Cells were untreated (Control) or infected with Hazara virus at MOI 1 for 1 h and maintained for 24 hpi (Virus). Total cell lysates were immunoprecipitated using the antibodies against CCHFV/Hazara (viral N). N-enriched immune complexes **(Left Panel)** and total cell lysates as a control **(Right Panel)** were analyzed by SDS-PAGE and stained with Coomassie Blue. Bands were excised and N-binding cellular proteins identified by in-gel digestion and LC-MS/MS analysis as shown in Table 1. The band indicated with a black arrow represent viral N. Displayed are representative gels from one of four independent experiments.

Since Hazara virus challenges resulted in modified epithelial migration (Figure 3) and reduced expression of several proteins important for cytoskeletal dynamics and homeostasis (Figures 4, 5), other parallel manipulations of cellular processes and programs might be of significance. We thus further aimed to identify partners of Hazara virus nucleocapsid protein (N) in the cellular proteome of human epithelia upon infection. To do so, epithelial monolayers were kept non-infected, or infected with Hazara virus at MOI 1 for 1 h, and at 24 hpi, total cell lysate samples were obtained and further immunoprecipitated using the antibodies against CCHFV/Hazara N. The N-enriched immune complexes, as well as total cell lysate samples were separated on SDS-PAGE and stained with Coomassie Blue (Figure 6). For the latter, no significant difference in protein patterns of infected and non-infected cells was observed (Figure 6, right panel). For the samples with N-enriched immune complexes, one additional protein band was reproducibly seen in the sample corresponding cells infected with Hazara virus (Figure 6, left panel).

Each gel line was cut into 10 slices, digested and analyzed by liquid chromatography and mass spectrometry (LC-MS/MS), where spectrum counts were used for quantitative comparisons of samples. To provide confidence, the experiments were repeated 4 times. The additional visible band detected in the sample corresponding cells infected with Hazara virus was identified as the viral N (Figure 6, left panel), which served as a control to confirm both infection and immunoprecipitation. Furthermore, 36 out of around 500 cellular proteins were typically identified in the N-enriched immune complexes and exhibited significant differences in affinity (Table 1).

**Table 1:** Alterations in the human cellular interactome of the Hazara virus N compared to the control non-infected cells.

**Identified proteins**	**Gene (Uniprot accession number)**	**Protein (Uniprot accession number)**	**MW kDa**	***P*-value**	**Fold change, (infected/control)**	**Functional groups of human proteins #**						
**PROTEINS DETECTED IN N-ENRICHED IMMUNE COMPLEXES ONLY IN VIRUS-INFECTED CELLS**												
Calnexin	CANX	P27824	72	0.0001	Detected only in virus-infected cells	**X**		**X**	**X**	**X**	**X**	**X**
Probable serine carboxypeptidase CPVL	CPVL	Q9H3G5	54	0.024			**X**					**X**
14-3-3 protein beta/alpha	YWHAB	P31946	28	0.025		**X**		**X**	**X**	**X**		**X**
Stress-induced-phosphoprotein 1	STIP1	P31948	68	0.025		**X**		**X**			**X**	**X**
LIM and SH3 domain protein 1	LASP1	Q14847	30	0.037						**X**		**X**
Dihydropyrimidinase-related protein 2	DPYSL2	Q16555	62	0.045						**X**		**X**
Ryanodine receptor 3	RYR3	Q15413	552	0.046					**X**	**X**		
Low-density lipoprotein receptor-related protein 1B	LRP1B	Q9NZR2	515	0.054				**X**				
Peroxiredoxin-1	PRDX1	Q06830	19	0.058		**X**	**X**	**X**		**X**		**X**
Protein disulfide isomerase BLOC1S5-TXNDC5	TXNDC5	Q86UY0	40	0.083				**X**			**X**	**X**
**PROTEINS DISPLAYED INCREASED BINDING TO N-ENRICHED IMMUNE COMPLEXES UPON VIRUS INFECTION**												
Hsc70-interacting protein	ST13	H7C3I1	16	0.0002	14						**X**	**X**
Fructose-bisphosphate aldolase A	ALDOA	P04075	45	0.013	19	**X**	**X**	**X**		**X**		**X**
F-box only protein 2	FBXO2	Q9UK22	33	0.028	2.8		**X**			**X**		**X**
60 kDa heat shock protein, mitochondrial, HSP60	HSPD1	P10809	61	0.029	2.2	**X**		**X**	**X**		**X**	**X**
Annexin A2	ANXA2	P07355	40	0.029	3.6	**X**			**X**	**X**		**X**
Malate dehydrogenase, cytoplasmic	MDH1	P40925	39	0.051	3.9		**X**	**X**		**X**		**X**
Phosphoglycerate kinase 1	PGK1	P00558	45	0.064	2.6		**X**			**X**		**X**
Villin-1	VIL1	P09327	93	0.066	2.4			**X**	**X**	**X**		**X**
Protein disulfide-isomerase A4	PDIA4	P13667	73	0.086	3.7	**X**		**X**	**X**	**X**	**X**	**X**
Regulation of nuclear pre-mRNA domain-containing protein 1B	RPRD1B	Q9NQG5	37	0.094	5.6	**X**				**X**		
Alpha-enolase	ENO1	P06733	47	0.095	2.1	**X**	**X**	**X**		**X**	**X**	**X**
Elongation factor 1-delta	EEF1D	P29692	69	0.097	2.4	**X**	**X**			**X**	**X**	
Transaldolase	TALDO1	P37837	38	0.1	3.1		**X**					**X**
**PROTEINS DISPLAYED DECREASED BINDING TO N-ENRICHED IMMUNE COMPLEXES UPON VIRUS INFECTION**												
Polyadenylate-binding protein	PABPC4	B1ANR0	68	0.0076	0.5	**X**						
Nucleolysin TIAR	TIAL1	Q01085	43	0.018	0.7	**X**						**X**
Non-POU domain-containing octamer-binding protein	NONO	Q15233	54	0.035	0.4	**X**		**X**		**X**		
Heterogeneous nuclear ribonucleoprotein Q	SYNCRIP	O60506	70	0.052	0.5	**X**						**X**
Stress-70 protein, mitochondrial, HSP70	HSPA9	P38646	74	0.052	0.7	**X**		**X**	**X**	**X**	**X**	**X**
ATP-dependent RNA	DDX3X	O00571	71	0.053	0.5	**X**		**X**	**X**	**X**		**X**
Histone H1.5	HIST1H1B	P16401	23	0.065	0.3	**X**			**X**			**X**
28S ribosomal protein S29, mitochondrial	DAP3	P51398	46	0.077	0.4	**X**				**X**		
RNA-binding protein EWS	EWSR1	Q01844	63	0.077	0.8	**X**						
Histone-arginine methyltransferase CARM1	CARM1	Q86X55	63	0.083	0.6	**X**				**X**		
Guanine nucleotide-binding protein subunit beta-2-like 1, Receptor of activated protein C kinase 1	GNB2L1	P63244	35	0.089	0.4	**X**	**X**			**X**		
Heterogeneous nuclear ribonucleoprotein L	HNRNL	P14866	64	0.091	0.7	**X**						**X**

*#Functional groups of human proteins: X, RNA and DNA processes; X, metabolism; X, cell defense and response to stress and pathogens; X, virus growth; X, cell morphology, migration, differentiation, proliferation and apoptosis; X, chaperone activity; X–membrane-bound vesicle communication. STRING and SCAFFOLD analyses were used to group proteins into functional classes. Data are from 4 independent experiments*.

Ten N-associated proteins were detected in virus-infected cells only, and 13 proteins displayed at least 2-fold increased binding to N-enriched immune complexes upon viral infection in comparison to the non-infected control (Table 1). These 23 proteins were considered as potential N partners in the Hazara infection. Twelve proteins displayed decreased binding to N-enriched immune complexes upon viral infection in comparison to non-infected control (Table 1). All 36 cellular proteins, typically identified in N-associated immune complexes and exhibited significant differences in affinity (Table 1), were included in the further bioinformatics analyses.

Bioinformatics analyses, using the search tool for the retrieval of interacting genes and proteins (STRING) and NCBI GO annotations by SCAFFOLD, were performed to investigate whether the identified N-associated cellular proteins could be placed into distinct functional classes. This revealed that they could be put in a groups being involved in: RNA and DNA processes, formation of membrane-bound vesicles, cell morphology, migration, differentiation, proliferation and apoptosis, metabolism, cellular defense and response to stress and pathogens, virus growth and chaperone activity (Table 1).

**Figure 7:**
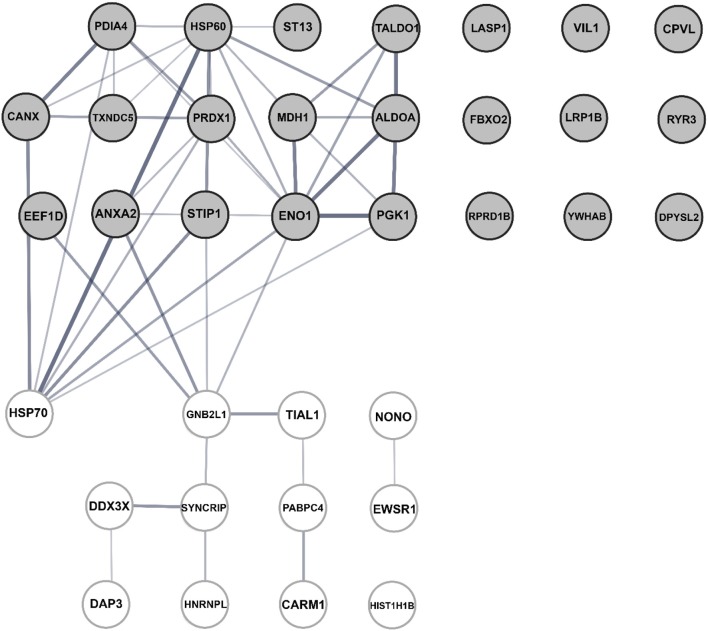
Bioinformatic assessment of the human cellular interactome of the Hazara virus N shown in Table 1. Network nodes represent human proteins pooled into two units. The upper unit with gray nodes represent 23 proteins suggested as potential N partners upon Hazara infection. These include 10 N-associated proteins detected only in virus-infected cells and 13 proteins displayed at least 2-fold increased binding to N-enriched immune complexes upon viral infection in comparison to non-infected control (Table 1). The lower unit with white nodes represent 12 proteins displayed decreased binding to N-enriched immune complexes upon viral infection in comparison to non-infected control (Table 1). Edges represent protein-protein interactions where line thickness indicates the strength of data support. The assessment is based on STRING analysis shown in Figure S1 and LC-MS/MS analysis and quantification of alterations in cellular proteome and interactome of N shown in Table 1.

Independent analyses of network interactions, using STRING, disclosed that out of the 36 host proteins, which were typically identified in N-enriched immune complexes, at least 24 were connected through distinct types of actions (Figure 7), as previously being established experimentally and from curated databases and predicted by gene neighborhood and co-occurrence or by protein co-expressions and homologies (Figure S5). The distinct, large node cluster of N-associated proteins having a rich network of protein-protein interactions (Figure 7) contained proteins with chaperone activity (Table 1, depicted in blue): calnexin, mitochondrial Hsp60 and Hsp70, stress-induced phosphoprotein 1, elongation factor 1-δ, α-enolase, hsc70-interacting protein and two members of the protein disulfide isomerase family. Other proteins with a rich network were ribosomal receptor of activated protein C kinase 1 and cytoplasmic heterogeneous nuclear ribonucleoprotein Q (Figure 7). Further bioinformatics analyses revealed clusters of proteins involved in RNA and DNA processes (Table 1, noted in red, Figure S6A), in membrane-bound vesicle communication (Table 1, shown in violet, Figure S6B), and in key cellular processes, such as cell morphology, migration, differentiation, proliferation and apoptosis (Table 1, shown in green).

Thus, epithelial cellular protein networks are strongly perturbed by a Hazara virus infection, where the viral structural N associates and interacts with an array of cellular proteins.

## Discussion

A fundamental feature of epithelial linings of multicellular organisms is their ability to repair wounds via regeneration and collective cellular movement (Friedl and Mayor, 2017). Both properties are crucial for the maintenance of the structural and functional integrity and homeostasis of tissues (Figure 1). Employing the model of epithelial wound healing (Figure 2), we show that the repair was perturbed upon Hazara virus infection (Figure 3). At early stages of infection, the migration rates of virus-infected cells were either similar to the control or even promoted (Figures 3A,B). This probably reflects the very short time for establishment of infection. Indeed, in CCHFV, RNA synthesis in the infected cells likely starts at 6–16 h after infection (Simon et al., 2009). Later, we observed consistently significantly decreased repair of the wounds, e.g., after longer presence of the virus (Figures 4, 5) and associated disturbances in the cellular proteome. During virus infection, a plethora of factors released from the cells may also have some impact on epithelial repair and barrier function (Bente et al., 2013).

Successful wound closure requires extensive remodeling of the cytoskeleton, which is controlled by a plethora molecules (Yang et al., 2009; Ivanov et al., 2010; Friedl and Mayor, 2017), and among them, scaffold IQGAP1 (Karlsson et al., 2012; Hedman et al., 2015) and AQP (Loitto et al., 2009; Karlsson et al., 2013), playing a pivotal role in regulation of cell migration, cytoskeleton dynamics, water homeostasis and vesicle communication. We therefore further focused our investigations on the mechanisms whereby Hazara virus could have on epithelial cell migration. Using confocal imaging and immunoblotting, we observed that the F-actin was disrupted and the expression and distribution of IQGAP1 and AQP6 were reduced upon Hazara infection (Figures 4, 5). This may allow virus to maintain long-term infections in the host and perturb the repair of epithelia by interfering with cytoskeletal structure and water homeostasis.

Our findings corroborate several reports on how microbial pathogens may manipulate signaling pathways of the host cells, particularly cytoskeletal dynamics, to help them invade, replicate and maintain infections (Chazal and Gerlier, 2003; Sewald et al., 2016). Thus, IQGAP1 regulates egress of Ebola virus (Lu et al., 2013), invasion and replication of Moloney leukemia virus (Leung et al., 2006) and virulence of swine fever virus (Gladue et al., 2011). Moreover, CCHFV did perturb strongly the microtubule and actin filaments during entry, growth and release to the surroundings (Andersson et al., 2004; Simon et al., 2009). Additionally, other studies have proposed a new role for AQP as important proteins during viral infections and inflammation. Thus, the levels of AQP1 and 5 were low during pulmonary, adenoviral infection (Towne et al., 2000). AQP4 was decreased in the acute phase of a Herpes simplex virus infection, but increased in the long-term of disease (Martinez Torres et al., 2007). An infection of human cells with CCHFV strain IbAR 10200 downregulated the AQP6 mRNA expression (Molinas et al., 2016). Furthermore, with Hazara virus as a model, we have recently provided an evidence for a protective role of AQP6 against virus infection (Molinas et al., 2016).

To further elucidate details on how the virus affects the cells, we aimed to identify potential partners of Hazara virus N in the epithelial cellular proteome upon infection. To do this, we employed immunoprecipitation, SDS-PAGE (Figure 6), LC-MS/MS (Table 1) and bioinformatics analyses (Figure 7). Upon virus infection, 36 out of around 500 cellular proteins were typically identified in N-enriched immune complexes and exhibited significant difference in in connectivity to viral N (Table 1). Among them, 10 proteins were detected only in virus-infected cells, and further 13 and 12 proteins displayed either increased or decreased binding to N-enriched immune complexes, respectively, upon viral infection in comparison to non-infected control (Table 1).

We identified proteins with chaperone activity (Figure 7, Table 1, noted in blue). They can interact with viral proteins, including N, and favor viral replication and block actin binding (Mirazimi et al., 1998; Horna-Terron et al., 2014; Abbas et al., 2015; Khachatoorian and French, 2016). Both Hsp60 and Hsp70, may also directly bind virus RNA (Nanda et al., 2004) and thereby control viral infection (Lahaye et al., 2012). Consistent with this scenario, Hsp70 association with *Nairovirus* N was required for viral replication (Surtees et al., 2016).

We also recognized the ribosomal receptor of activated protein C kinase 1 and cytoplasmic heterogeneous nuclear ribonucleoprotein Q (Figure 7), earlier being implicated in viral replication and release (Liu et al., 2009; Demirov et al., 2012).

A cluster of the proteins is involved in RNA and DNA processes (Table 1, noted in red). Viral N has several RNA binding domains that facilitate ribonucleoprotein formation in mature virions. This should also give a basis for molecular mimicry allowing N to associate with host cellular RNA-binding proteins, to be transported to the nuclear region and to utilize host protein machinery for virus production (Rowland and Yoo, 2003; Wulan et al., 2015).

A group of N-binding proteins orchestrates membrane-bound vesicle communication (Table 1, shown in violet). Extracellular membrane vesicles (EMV) are membrane-bound structures shedding from cells into the environment and playing a role in cell-cell communication through protein, lipid and nucleic acid transfer. Both pathogenic bacteria and viruses can hijack host EMV and thereby enhance their pathogenicity (Schwab et al., 2015; Turkina et al., 2015). Coxsackie virus disseminates within host EMV upon stem cell migration and differentiation and thereby spreads to new cells (Robinson et al., 2014). Severe fever with thrombocytopenia syndrome virus (SFTS), which is a new *Bunyaviridae* family member, incorporates itself into EMV and use them as a delivery system for its own spreading (Silvas et al., 2016). We suggest that Hazara virus may also take the advantage of host EMV to promote spreading through cell-to-cell contacts and thereby the establishment and progression of infection.

A large group of proteins is required for regulation of cell morphology, migration, differentiation, proliferation and apoptosis (Table 1, shown in green). Indeed, virus can target and manipulate host cytoskeleton dynamics and organization which may ensure virus replication and spread (Sewald et al., 2016). Dendritic cells (Cunningham et al., 2010), monocytes (Daley-Bauer et al., 2014), and T-cells (Murooka et al., 2012) can be hijacked by varicella zoster virus, cytomegalovirus and HIV, and thereby used as migratory vehicles for viral dissemination.

In many RNA viruses, N is known not only as a structural but also as a functionally important protein. Thus, N binds to the viral RNA genome and forms ribonucleoproteins in mature virions, which is seen in severe acute respiratory syndrome coronavirus (Huang et al., 2004), infectious bronchitis virus (Spencer and Hiscox, 2006), and CCHFV (Morikawa et al., 2007). Viral N interferes with immune system to enhance virus virulence, arrest host cell cycle and inactivate chaperons (Emmott et al., 2013; McBride et al., 2014). By such a broader interaction with host cell proteins, directly or in-directly, N may manipulate host cytoskeleton dynamics and other distinct cellular processes that potentially affect the virus life cycle.

**Figure 8:**
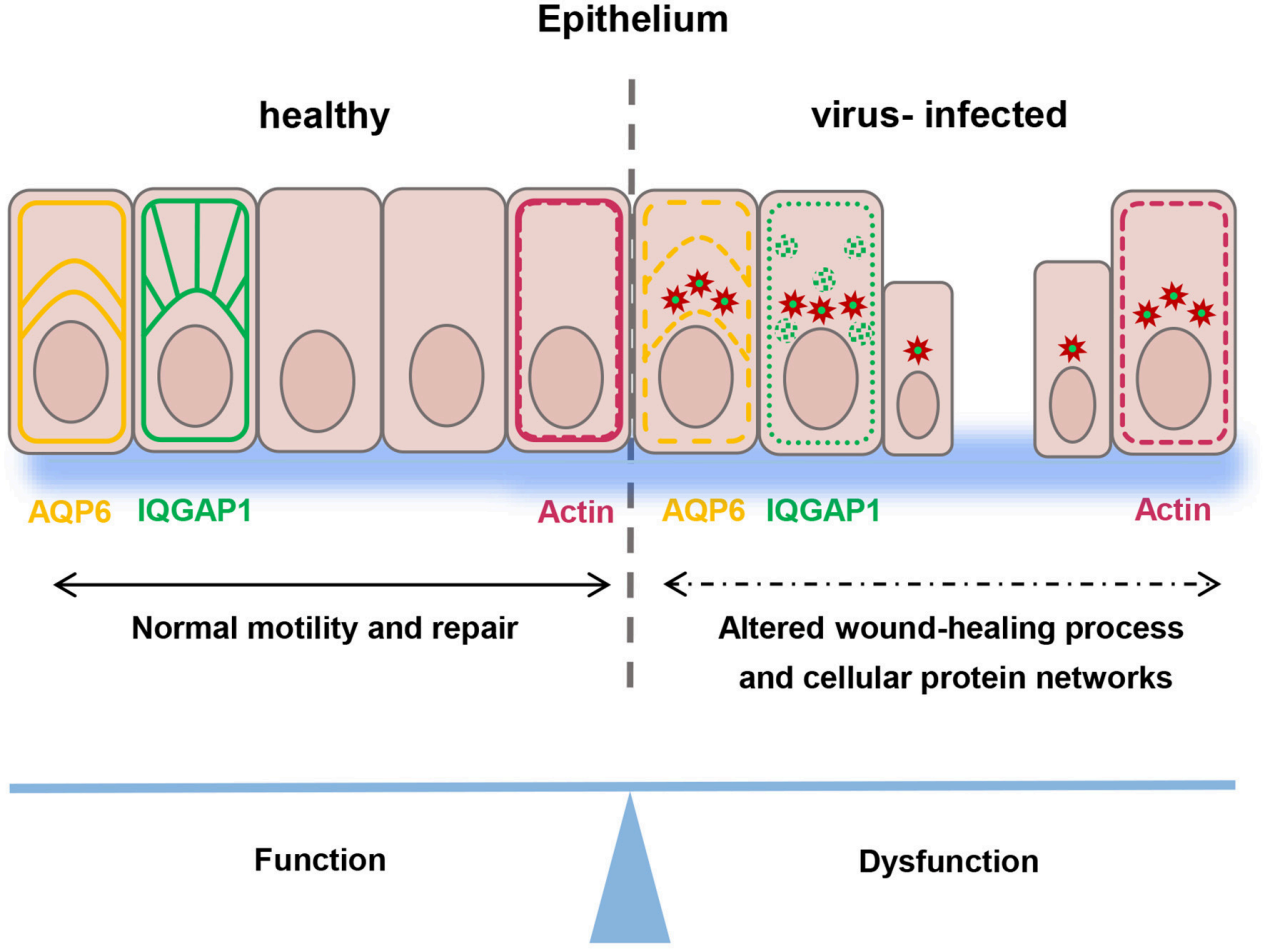
Schematic model. Epithelial wound healing process and cellular protein networks (shown as arrows) in human epithelial cells (in pink) are perturbed by Hazara virus infection. This is paralleled by disruption in the organization of actin cytoskeleton (in red) and reduction of the expression and distribution of IQGAP1 (in green) and AQP6 (in yellow), events that influence homeostasis in the intestine and along blood vessel walls and integrity of an individual.

In summary, our study demonstrates that Hazara virus can modulate migration of human epithelial cells, disrupt their actin cytoskeleton organization, cellular distribution and reduce the expression of IQGAP1 and AQP6, which help regulate cytoskeleton dynamics and water homeostasis. Moreover, upon infection, Hazara virus structural N is engaged with an array of cellular protein interactions (Figure 8). Our interactome data provide a foundation for future work on mechanistic insights into biology of an infection. Taken together, these events interfere with many cellular processes that potentially facilitate the virus life cycle, but they are also deleterious to cell renewal and wound healing at epithelial and endothelial linings being so essential to organism homeostasis under normal physiological situation and after bacterial or virus injury and infection.

## Author contributions

EV conceived and designed the experiments; EV, MT, and AM performed the experiments; EV, MT, AM, and K-EM contributed to the data analysis and interpretation of the results; AliM contributed materials; EV drafted the manuscript; EV, MT, AM, and K-EM edited the manuscript; All authors approved the final version of the manuscript.

## Conflict of interest statement

The authors declare that the research was conducted in the absence of any commercial or financial relationships that could be construed as a potential conflict of interest. The reviewer TF and handling Editor declared their shared affiliation, and the handling Editor states that the process nevertheless met the standards of a fair and objective review.

## Supplementary material

Figure S1 Visualization of Hazara virus, F-actin, IQGAP1 and AQP6 in epithelial cells. Cells were untreated (Control) or infected with Hazara virus at MOI 1 for 1 h and maintained for 24 hpi. Samples were then fixed and stained for: **(A)** viral N (red) and F-actin (green); **(B)** viral N (red) and IQGAP1 (green); **(C)** viral N (green) and AQP6 (red); nuclei were labeled with DAPI (blue). Samples were analyzed by confocal microscopy. The data is from one representative of three independent experiments. Image size is 67.6 × 67.6 μm and pixel size is 0.13 μm. Quantification of immunofluorescence intensity profiles for F-actin **(D)**, IQGAP1 **(E)** and AQP6 **(F)** measured across the cell monolayers as indicated by blue arrows (control) and yellow arrows (virus-infected cells, MOI 1, 24 hpi). The length of arrows reflects the distance of 60 μm.

Figure S2 Additional quantification of F-actin fluorescence intensity profiles. The set of experiments were performed and quantification of fluorescence intensity profiles were done as in Figure 4A and Figures S1A,D. Non-infected cells are indicated by blue arrows (control) and virus-infected cells are coded by yellow arrows (MOI 1, 24 hpi). The length of arrows reflects the distance of 60 μm. Shown are F-actin intensity profiles measured across the cell monolayers from 6 representative cell regions and three independent experiments performed on separate days from different cell passages.

Figure S3 Additional quantification of IQGAP1 fluorescence intensity profiles. The set of experiments were performed and quantification of fluorescence intensity profiles were done as in Figure 4B and Figures S1B,E. Non-infected cells are indicated by blue arrows (control) and virus-infected cells are coded by yellow arrows (MOI 1, 24 hpi). The length of arrows reflects the distance of 60 μm. Shown are IQGAP1 intensity profiles measured across the cell monolayers from 6 representative cell regions and three independent experiments performed on separate days from different cell passages.

Figure S4 Additional quantification of AQP6 fluorescence intensity profiles. The set of experiments were performed and quantification of fluorescence intensity profiles were done as in Figure 4C and Figures S1C,F. Non-infected cells are indicated by blue arrows (control) and virus-infected cells are coded by yellow arrows (MOI 1, 24 hpi). The length of arrows reflects the distance of 60 μm. Shown are AQP6 intensity profiles measured across the cell monolayers from 6 representative cell regions and three independent experiments performed on separate days from different cell passages.

Figure S5 Bioinformatic STRING analysis of the human cellular interactome of the Hazara virus N shown in Table 1. Colored network nodes represent query proteins. Edges represent protein-protein interactions and include different type of actions depicted by the colored lines. For known interactions: pink, experimentally determined; turquoise, from curated databases. For predicted interactions: green, gene neighborhood; blue, gene co-occurrence. For others interactions: olive green, literature mining; black, co-expression; purple, protein homology.

Figure S6 Additional STRING bioinformatic analysis of the human cellular interactome of the Hazara virus N shown in Table 1. Network nodes represent proteins. Red nodes, query proteins and first shell of interaction for those involved in **(A)** RNA and DNA processes and those associated to **(B)** membrane bond vesicles. White nodes, second shell of interaction. Edges represent protein-protein interactions and include different type of actions depicted by the colored lines as in Figure S1.
