# Supplementary figures and images for: Perturbation of Wound Healing, Cytoskeletal Organization and Cellular Protein Networks during Hazara Virus Infection
Source: Front Cell Dev Biol. 2017 Nov 21;5:98. doi: 10.3389/fcell.2017.00098 (PMC5702460; doi:10.3389/fcell.2017.00098)

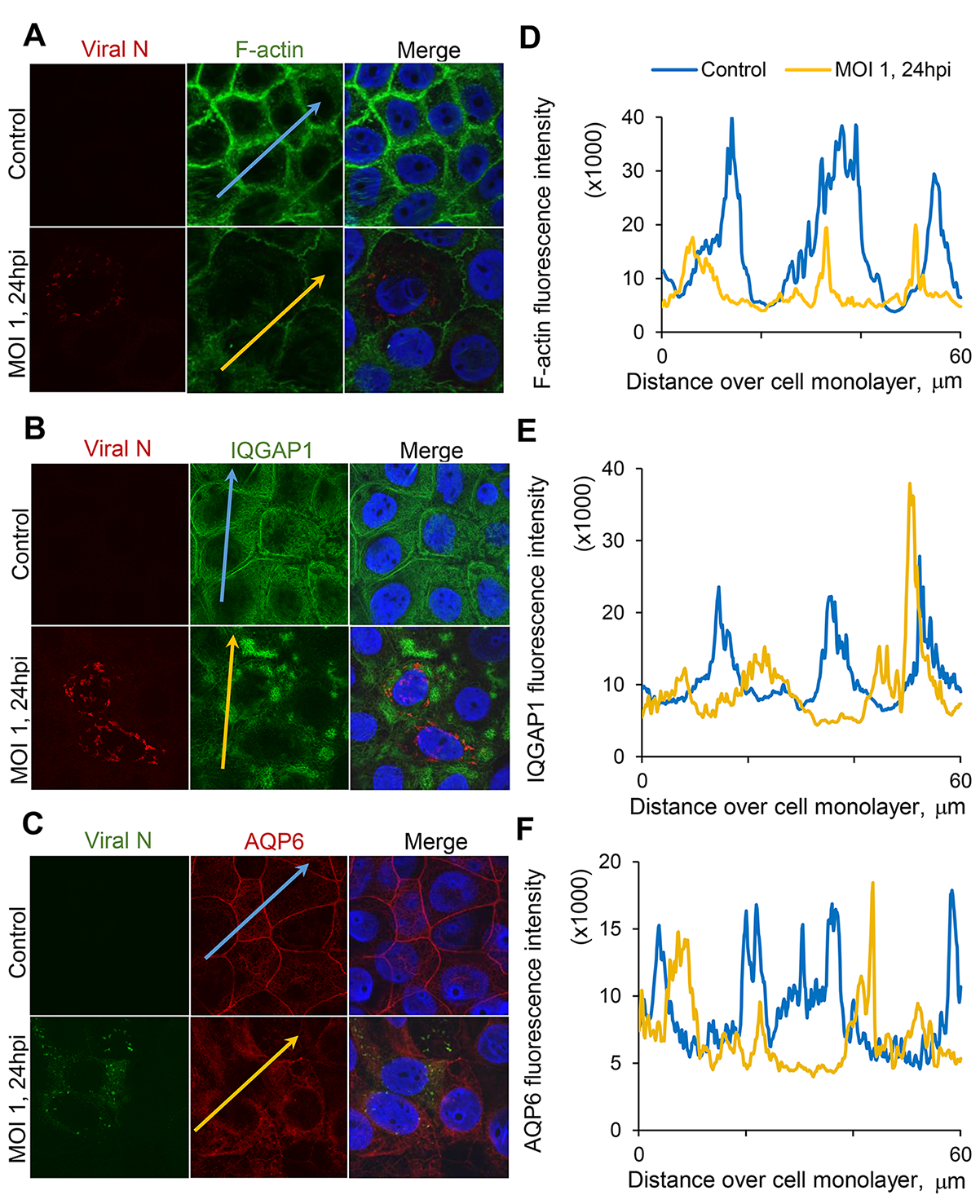

Supplement: Figure S1 — Visualization of Hazara virus, F-actin, IQGAP1 and AQP6 in epithelial cells. Cells were untreated (Control) or infected with Hazara virus at MOI 1 for 1 h and maintained for 24 hpi. Samples were then fixed and stained for: (A) viral N (red) and F-actin (green); (B) viral N (red) and IQGAP1 (green); (C) viral N (green) and AQP6 (red); nuclei were labeled with DAPI (blue). Samples were analyzed by confocal microscopy. The data is from one representative of three independent experiments. Image size is 67.6 × 67.6 μm and pixel size is 0.13 μm. Quantification of immunofluorescence intensity profiles for F-actin (D), IQGAP1 (E) and AQP6 (F) measured across the cell monolayers as indicated by blue arrows (control) and yellow arrows (virus-infected cells, MOI 1, 24 hpi). The length of arrows reflects the distance of 60 μm. [file Image1.TIF]

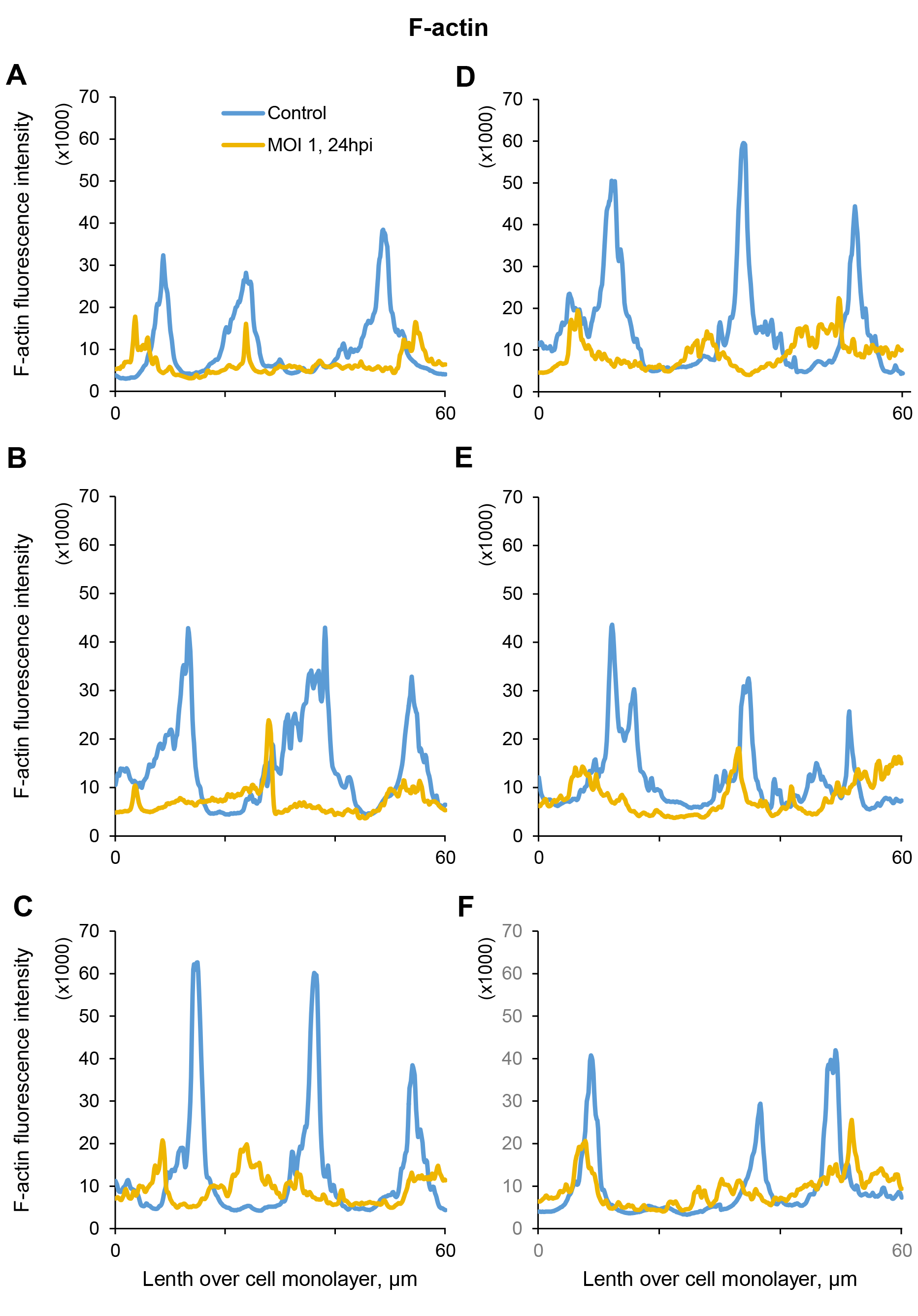

Supplement: Figure S2 — Additional quantification of F-actin fluorescence intensity profiles. The set of experiments were performed and quantification of fluorescence intensity profiles were done as in Figure 4A and Figures S1A,D. Non-infected cells are indicated by blue arrows (control) and virus-infected cells are coded by yellow arrows (MOI 1, 24 hpi). The length of arrows reflects the distance of 60 μm. Shown are F-actin intensity profiles measured across the cell monolayers from 6 representative cell regions and three independent experiments performed on separate days from different cell passages. [file Image2.TIF]

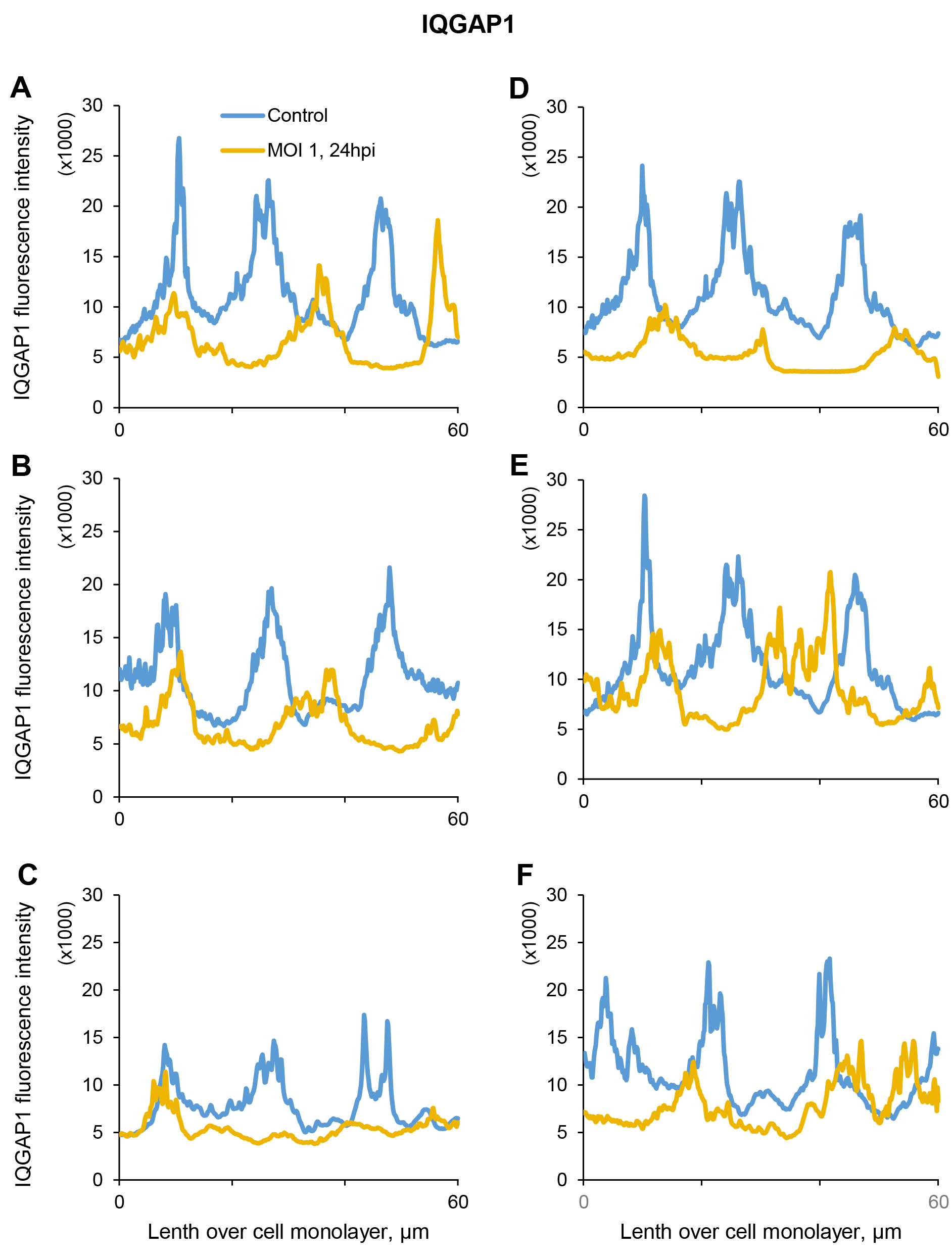

Supplement: Figure S3 — Additional quantification of IQGAP1 fluorescence intensity profiles. The set of experiments were performed and quantification of fluorescence intensity profiles were done as in Figure 4B and Figures S1B,E. Non-infected cells are indicated by blue arrows (control) and virus-infected cells are coded by yellow arrows (MOI 1, 24 hpi). The length of arrows reflects the distance of 60 μm. Shown are IQGAP1 intensity profiles measured across the cell monolayers from 6 representative cell regions and three independent experiments performed on separate days from different cell passages. [file Image3.TIF]

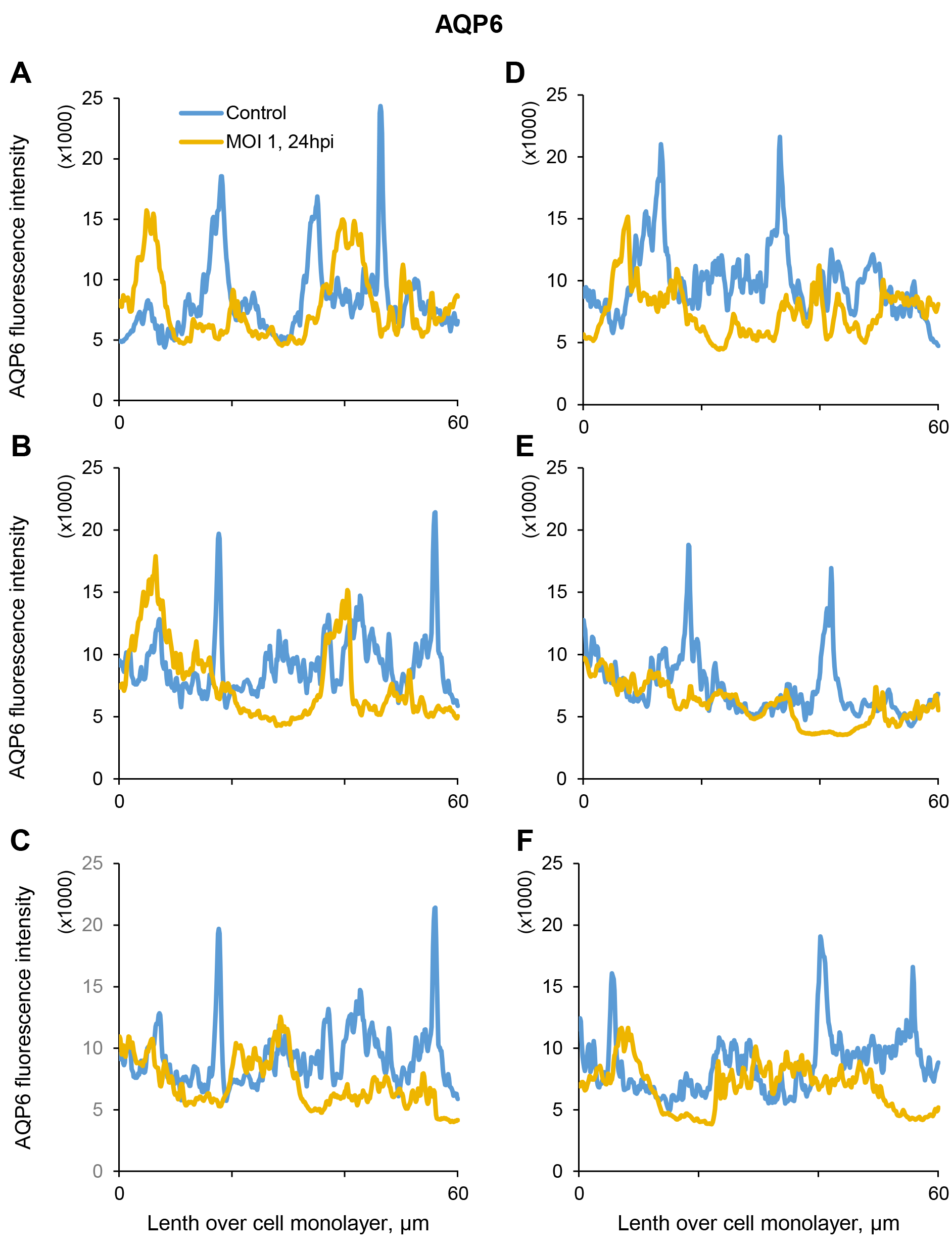

Supplement: Figure S4 — Additional quantification of AQP6 fluorescence intensity profiles. The set of experiments were performed and quantification of fluorescence intensity profiles were done as in Figure 4C and Figures S1C,F. Non-infected cells are indicated by blue arrows (control) and virus-infected cells are coded by yellow arrows (MOI 1, 24 hpi). The length of arrows reflects the distance of 60 μm. Shown are AQP6 intensity profiles measured across the cell monolayers from 6 representative cell regions and three independent experiments performed on separate days from different cell passages. [file Image4.TIF]

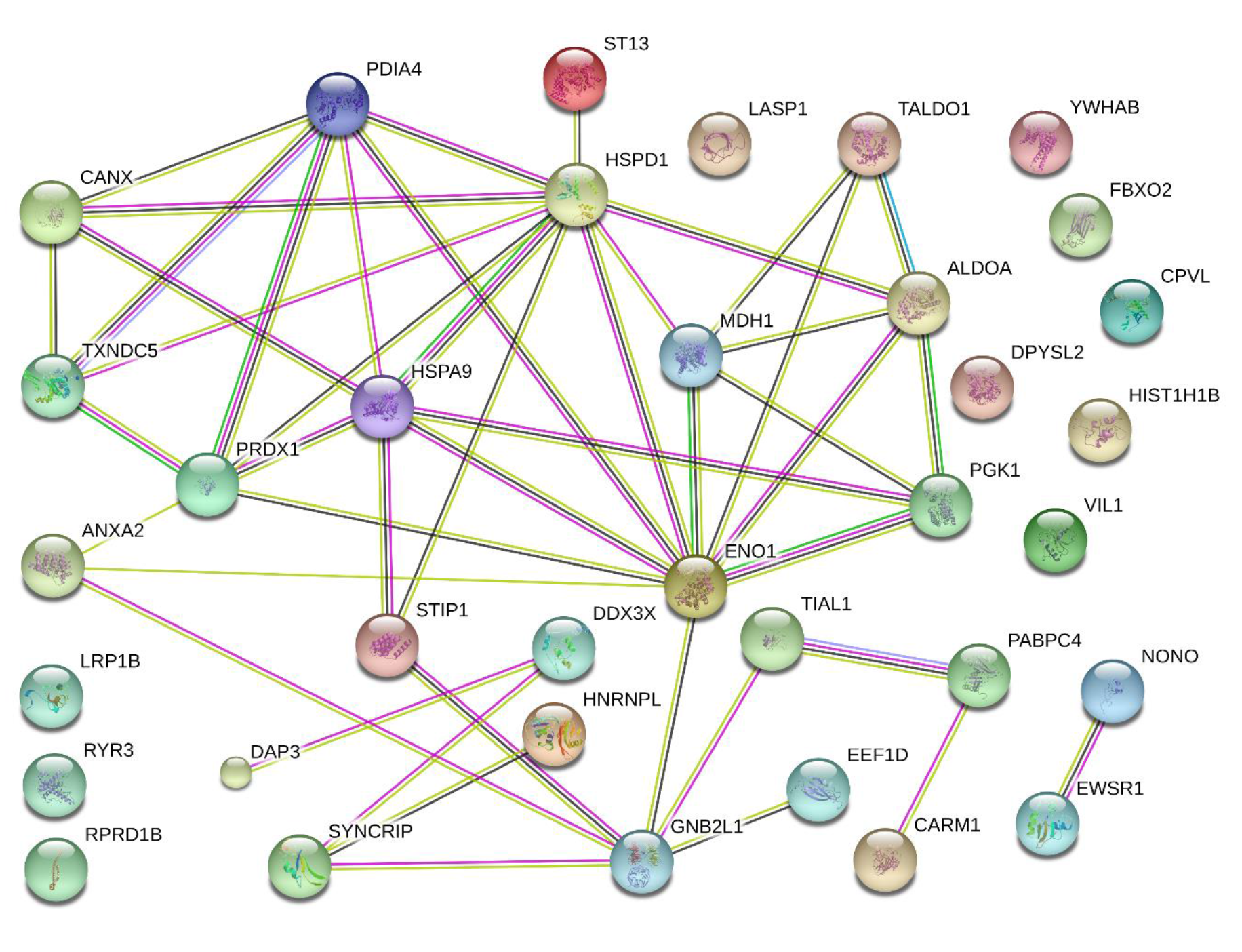

Supplement: Figure S5 — Bioinformatic STRING analysis of the human cellular interactome of the Hazara virus N shown in Table 1. Colored network nodes represent query proteins. Edges represent protein-protein interactions and include different type of actions depicted by the colored lines. For known interactions: pink, experimentally determined; turquoise, from curated databases. For predicted interactions: green, gene neighborhood; blue, gene co-occurrence. For others interactions: olive green, literature mining; black, co-expression; purple, protein homology. [file Image5.TIF]

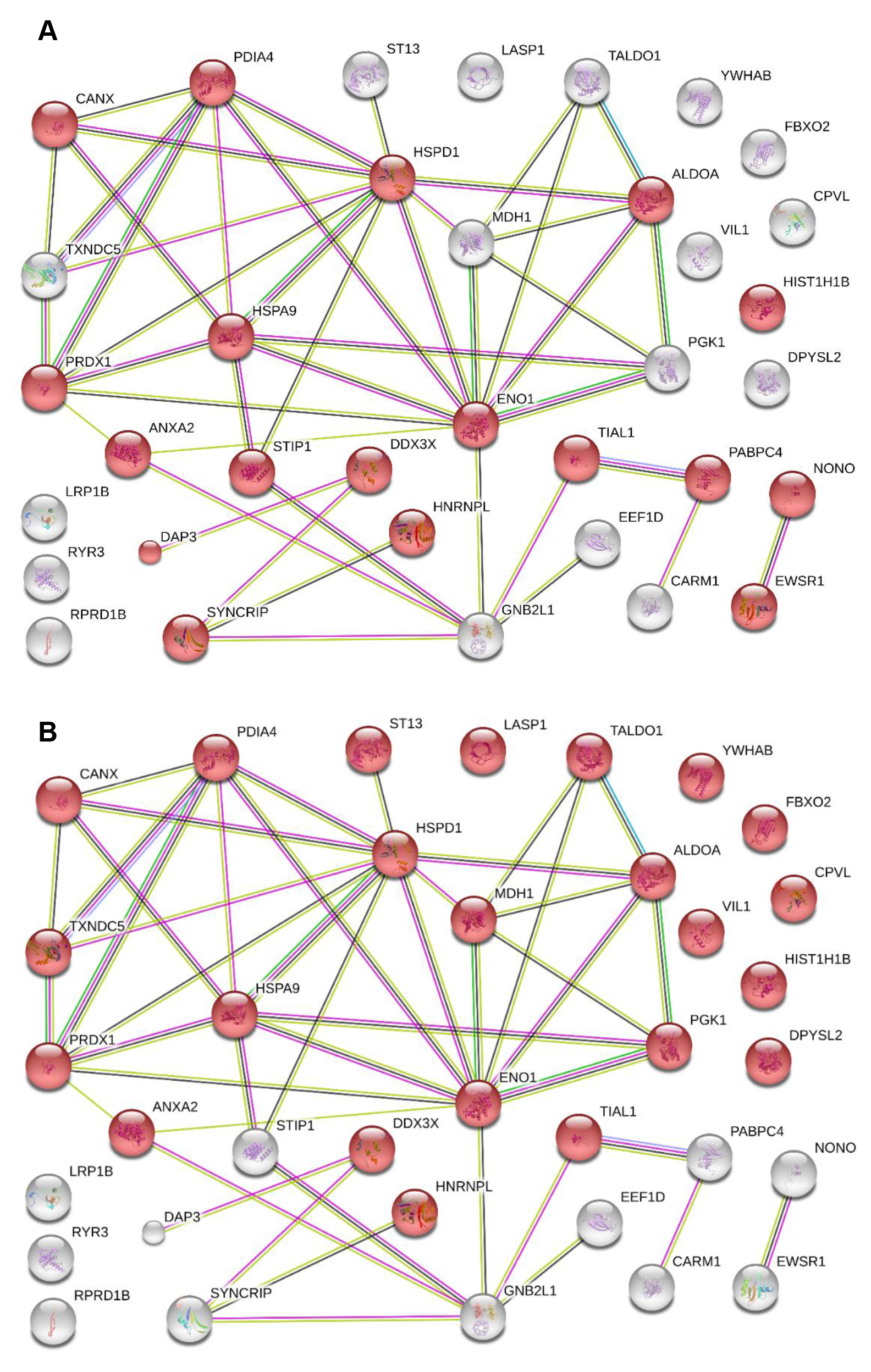

Supplement: Figure S6 — Additional STRING bioinformatic analysis of the human cellular interactome of the Hazara virus N shown in Table 1. Network nodes represent proteins. Red nodes, query proteins and first shell of interaction for those involved in (A) RNA and DNA processes and those associated to (B) membrane bond vesicles. White nodes, second shell of interaction. Edges represent protein-protein interactions and include different type of actions depicted by the colored lines as in Figure S1. [file Image6.TIF]
